# Cytomolecular Organisation of the Nuclear Genome

**DOI:** 10.3390/ijms232113028

**Published:** 2022-10-27

**Authors:** Robert Hasterok, Natalia Borowska-Zuchowska, Ewa Robaszkiewicz

**Affiliations:** Plant Cytogenetics and Molecular Biology Group, Institute of Biology, Biotechnology and Environmental Protection, Faculty of Natural Sciences, University of Silesia in Katowice, 40-032 Katowice, Poland

Modern molecular cytogenetics allows many aspects of the nuclear genome structure, function, and evolution to be analysed within the topographic context of mitotic and meiotic chromosomes and interphase nuclei. It also allows high-resolution mapping in such substrate as extended chromatin fibres. These major advances have been made possible by robust methodologies. For example, DNA:DNA fluorescence in situ hybridisation (FISH) supported by state-of-the-art microscopes enables a two- or three-dimensional multicolour visualisation of highly repetitive, low-repeat, and single-copy sequences in the genome. Antibodies conjugated with fluorescent labels raised against specific targets can be used to selectively localise the epigenetically modified components of chromatin, such as methylated DNA and histones. They also may be used to highlight the proteins forming important yet complex biological structures, such as the synaptonemal complex and the enzymatic machinery responsible for the genetic recombination events during meiosis.

This Special Issue “Cytomolecular Organisation of the Nuclear Genome” consists of 14 peer-reviewed publications, 12 articles (original papers), and two reviews. Together, they provide insight into the recent advances in cytomolecular analyses in diverse eucaryote groups, including animals such as birds, fishes, and humans, and plants—wild species, crops, and some model plants.

One of the fundamental aims of cytogenetic analyses is the unambiguous identification of all chromosomes in the complement. In rare cases, this is feasible based solely on the morphometric analyses of chromosomes. However, other chromosomal landmarks are required in the vast majority of species. The gold standard is chromosome painting (CP) which is based on FISH with probes that hybridise with the entire chromosomes or their specific segments in a chromosome-specific manner. CP enables the precise tracking of individual chromosomes at any stage of the cell cycle and provides invaluable information regarding their structure and evolution, as well as their arrangement and behaviour during the interphase. Due to significant differences in the genome size and repetitive DNA content and distribution, CP has long been used for the study of chromosomes in vertebrates, mammals, and birds in particular, e.g., in [1,2]. However, in the past, its application in plants was methodologically much more complicated as it required creating libraries of genomic DNA fragments cloned in high-capacity vectors, such as bacterial artificial chromosomes (BACs). To avoid unwanted cross-hybridisation between the probes, only the pools of the BACs containing low-repeat sequences were used as probes in the BAC-FISH experiments. For many years, these factors limited the application of CP in plants to a handful of small-genome species, usually model plants, such as *Arabidopsis thaliana* and its relatives in dicots, e.g., in [3], and representatives of the *Brachypodium* genus in monocots, e.g., in [4]. However, recent advances in the use of single-copy oligonucleotides as probes (oligo-FISH) has made CP applicable to virtually any plant, including previously intractable large-genome grasses, like *Avena* [5], and many Triticeae [6]. Thus, in the first review of this Special Issue, Liu and Zang [7] provide a comprehensive overview of the construction and application of the oligo-FISH probes. They first consider the various aspects of these probes’ design, such as oligonucleotide length, their thermodynamic properties, and the specificity. Then, they focus on a detailed characterisation and evaluation of the various oligo probe design tools and web platforms. Amongst their conclusions, the authors underline the importance of choosing an optimal tool for the oligo-probes design and the necessity of considering factors such as plant versus animal material, the research objective of planned FISH analyses, and probe-target hybridisation stability.

In another review, Knytl et al. [8] focus on the teleost fish genus *Carassius*, which includes taxa that differ in their phylogeny, sex determination, reproduction, and ecology. After familiarising the reader with the taxonomy of this fish genus, the authors provided an overview of karyotyping using a morphometrical approach. They also considered more advanced cytogenetic analyses involving chromosome banding techniques and FISH with 45S and 5S ribosomal DNA (rDNA) as probes. Then, they pointed to such aspects of *Carassius* reproduction as sexuality, unisexuality, and asexuality followed by variability in the chromosome numbers and ploidy levels, where an insight was gained using FISH with genomic DNA probes (GISH) or CP to characterise the interspecific hybridisation events in this genus. They discussed the importance of gynogenetic reproduction for the rapid expansion and invasiveness of the polyploid *Carassius* taxa, ending with an overview of the cytogenetic analyses of the sex chromosomes, which provided some insight into the complicated issue of sex determination in these fishes. They conclude that cytogenetic investigations into this genus can help us to understand the complex evolution of the *Carassius* genus and to protect its endangered representatives, such as *C. carassius*.

In the first article, Siminikova et al. [9] analyse the genome structure at the chromosomal level in the genus *Musa*, which includes edible bananas. Using oligo-FISH with arm-specific painting probes, they compared 20 representatives of the section Eumusa, including the subspecies of the wild *M. acuminata* and *M. balbisiana* and their inter- and intraspecific hybrids. They defined the differences in the chromosome structure that allowed the cytogenetic discrimination of individual accessions. This also enabled the authors to suggest the putative progenitors of cultivated bananas and gave an insight into the genomic constitution and evolution of aneuploid banana clones. These findings are likely to aid breeding programmes while selecting the optimal parents for hybridisation.

In the next article, del Priore and Pigozzi [10] study the relationship between the synaptonemal complex (SC) and attached DNA, regarding the crossover rates at prophase I of meiosis in chicken oocytes. Using immunolocalisation and FISH with chromosome-specific BAC clones, the authors demonstrated the link between the SC/DNA ratios and recombination frequencies observed between the various segments of a given chromosome and between the different chromosomes in a karyotype. In microbivalents and in the terminal segment of the largest macrobivalent, higher crossover rates were correlated with the presence of SCs longer than expected based on the amount of attached DNA. Since the SC-DNA loop organisation is evolutionarily conserved, the authors suggest that their findings can also be applied to other sexually reproducing organisms.

The article by Liaw et al. [11] describes the research on the epigenetic interactions between the DNA originating from evolutionarily distant organisms. A ~30 Mb long fragment of *A. thaliana* DNA in a human–plant hybrid cell line was the object of the study. Using differential methylation analysis, they revealed that some plant DNA methylation could be maintained in a highly different genomic background of the hybrid cell line even after 300 days. An expression analysis demonstrated that six of the 10 *A. thaliana* genes were still active in 60- and 300-day-old hybrid cell lines, but no relation was found between their expression and function. Though this study currently extends the understanding of interactions between the utterly diverse genomes primarily in terms of fundamental research, it may also have potential in future biotechnological applications.

As per their article, Kudryavtseva et al. [12] describe and discuss the use of tyramide FISH (Tyr-FISH) to enable the mapping of the short targets in plant mitotic chromosome preparations. In the Results, the authors focused on such methodological aspects as probe preparation and labelling, with special attention being put on increasing the signal–noise ratio and reducing the background, chromosome preparation, and quenching the horseradish peroxidase. Then, they described the dual-colour visualisation of two specific amplicons, 3.2 kb and 1.6 kb long, respectively, followed by the alignment of cytogenetic and genetic maps. Some further methodological and applicational aspects are raised in the Discussion, while the Materials and Methods consist of a step-by-step protocol. The key conclusion is that even in the age of high-throughput next-generation sequencing approaches, FISH can be a valuable tool for physical mapping, and Tyr-FISH, with its hypersensitive detection, may support the validation and integration of the sequencing data.

Sajid et al. [13] investigate the 3D chromosome arrangement at the prophase in human lymphocytes in the following paper. Using an advanced imaging technique known as serial block-face scanning electron microscopy, they identified all the chromosomes based on the compact axial structure within the chromosome core. Moreover, the authors provided novel information regarding the relative positioning of all chromosomes within a single prophase nucleus. Thus, this first 3D human prophase karyotype provides novel information concerning the spatial positioning of the chromosomes at the onset of mitosis. However, an open question remains whether the observed arrangement is universal or tissue/organ specific. Extending this study will be important to fundamental research and clinical diagnostics.

The paper by Yu et al. [14] is another spectacular example showing the potential of oligo-FISH in the advanced karyotyping of plant chromosomes. The authors used maize chromosome painting probes in sequential FISH experiments to comparatively analyse chromosomes in sorghum and *Tripidium arundinaceum*, which allowed all chromosomes of these two species to be identified, in the case of *T. arundinaceum*, for the first time. This is of considerable importance as the latter species is an important wild resource for sugarcane. Thus, the possibility of discriminating the individual chromosomes of *T. arundinaceum* should benefit trait improvement in sugarcane breeding.

In the next article, Sochorova et al. [15] provide a major update on the rDNA loci database (https://www.animalrdnadatabase.com/ (accessed on 7 October 2022)). Before its original release in 2016, the cytogenetic information on the number and chromosomal localisation of these loci was dispersed across the literature, but now this database provides such information in a user-friendly and easy-to-access manner. The current release, 2.0, contains the data regarding 2800 animal species belonging to 340 families, gathered from 1040 publications. When analysing the database contents, the authors focused on such aspects as a variation in the numbers of the rDNA loci, the occurrence of these loci in chromosomes with a limited or no recombination capacity, and the relationship between the numbers and the activity of the 45S rDNA loci. Although analysing the database content already allows some promising observations to be made, according to the authors, it currently only contains information for about less than 0.2% of the species. Thus, to make the data more complete and any metanalyses more meaningful, information about the rDNA loci organisation in the less-represented groups needs to be supplied.

Bacovsky et al. [16] present a study on mitosis in *Silene latifolia* and *S. dioica*, paying particular attention to the large heteromorphic X and Y sex chromosomes in these dioecious species. The authors tracked the position and behaviour of the sex chromosomes from the metaphase to telophase using FISH with the STAR-C centromeric and X43.1 subtelomeric repeat probes and 2-D/3-D microscopy. They measured the extension of the spindle axis in telophase and estimated the upper limit of the longest chromosomal arm (Yq) expansion. They suggested that although it can be species-specific, the Y chromosome expansion in these dioecious *Silene* species can generally be limited by the spindle axis extension. This study also revealed that the distant positioning of the sex chromosomes from the central interpolar axis compensates for their movement. Thus, this study contributes to a better understanding of the integrity of mitosis in plants with large heteromorphic X and Y sex chromosomes.

The article by Senderowicz et al. [17] provides a comprehensive study on the chromosomal organisation and evolution of rDNA loci in the complex and taxonomically challenging genus *Crepis* in the Asteraceae family. Using fluorescent bandings, FISH with rDNA probes and nr-ITS-based phylogenetic analyses, the authors determined the number and chromosomal location of 5S and 35S rDNA loci, reconstructed their ancestral numbers within the genus, and inferred the patterns of their evolution. Based on 5S rDNA non-transcribed spacer sequence analyses, they also provided some insight into both the inter- and intraspecific polymorphism of these genes. One of the major conclusions is that more detailed studies of *Crepis* genomes should involve chromosomal markers representing repetitive DNA families other than rDNA.

In the following article, Ramirez et al. [18] analyse the karyotype of *Solea senegalensis* (Senegalese sole), one of the aquaculturally most important flatfish species. Using BAC-FISH, the authors constructed a detailed cytogenetic map that they then integrated with already published linkage and physical maps. They also performed a comparative synteny analysis of each *S. senegalensis* chromosome with two other flatfish species used as the references. In addition, the repeatome of *S. senegalensis* was analysed as a potential contributor to the evolution of biarmed chromosomes 1–9. The key conclusion is that differences in the repeat arrangements in the chromosomes remodelled during the evolution may suggest some role that these sequences play in the rearranged regions.

Bara-Halama et al. [19] is the first study in plants that provides some in situ insight into the epigenetic status of specific DNA sequences in micronuclei. They used *B. distachyon*, part of the model grass genus *Brachypodium* (see a recent comprehensive review [20] for more detail). Its well-developed genomic, e.g., in [21,22] and cytogenetic resources, e.g., in [4,23,24] also make this genus an amenable model of advanced and extensive cytomolecular analyses of the chromosome and karyotype structure and evolution, e.g., in [25,26,27,28,29], interphase nucleus organisation in [30], selective rDNA inactivation (nucleolar dominance), e.g., in [31,32,33], and genome stability after an induced mutagenesis, e.g., in [34,35]. In their current research [19], the authors applied a sequential immunolocalisation of 5-methylcytosine and FISH with 5S and 25S rDNA probes to study the material subjected to a mutagenic treatment using maleic acid hydrazide. They observed several kinds of micronuclei at various frequencies, which differed in their 5S rDNA and 35S rDNA composition, and the methylation status of the foci occupied by these sequences. One of the conclusions and future perspectives is that it is worthwhile to determine whether there is any link between the DNA methylation patterns of micronuclei and their further fate during the cell cycle.

In the final publication of this Special Issue, Yucel et al. [36] show the comparative analyses of some aspects of karyotype organisation in the two subgenera of *Onobrychis* (sainfoins). This legume genus contains numerous species, some of them economically valuable as forage or even crops, whose phylogenetic relations are complex and not fully resolved. Using FISH, the authors determined the number and chromosomal distribution of 35S and 5S rDNA sites and revealed them to be similar and stable in polyploids, but highly polymorphic among diploids of this genus. Based on the analyses of nuclear ribosomal internal transcribed spacer (nrITS) sequences, they also reconstructed phylogenetic relationships, which was a prerequisite to inferring the ancestral states of the chromosome number and 35S and 5S rDNA loci number. It allowed the events and mechanisms responsible for shaping both the chromosome numbers and the numbers and chromosomal distributions of rDNA loci in *Onobrychis* to be hypothesised. This study represents a solid foundation for future, more advanced cytomolecular analyses.

Recent years have brought unprecedented advances in sequencing technologies, which have become faster, cheaper, and more potent than ever before. In contrast, molecular cytogenetic approaches are methodologically arduous, sometimes recalcitrant, low-throughput, and reluctant to automation. Yet, as demonstrated in the publications contributing to this Special Issue, despite those ‘inborn’ features, modern molecular cytogenetics provides a helpful link between cytology and genomics. These facts encourage us to continue as the Topical Collection on “Cytomolecular Organisation of the Nuclear Genome”: https://www.mdpi.com/journal/ijms/topical_collections/3UE88980UX (accessed on 7 October 2022), which we hope will be attractive to both the contributors and readers.

## Data Availability

Not applicable.
